# Periorbital and Central Nervous System Infection Due to *Arcanobacterium haemolyticum*: Case Report and Review of the Literature

**DOI:** 10.3390/microorganisms13092208

**Published:** 2025-09-20

**Authors:** Pierangelo Chinello, Alessandro Capone, Samir Al Moghazi, Paolo Cirillo, Carla Fontana, Stefania Cicalini

**Affiliations:** 1 Systemic and Immune Depression-Associated Infections Unit, National Institute for Infectious Diseases “Lazzaro Spallanzani”, IRCCS, 00149 Rome, Italy; alessandro.capone@inmi.it (A.C.); samir.almoghazi@inmi.it (S.A.M.); paolo.cirillo@inmi.it (P.C.); stefania.cicalini@inmi.it (S.C.); 2 Microbiology and Biobank Unit, National Institute for Infectious Diseases “Lazzaro Spallanzani”, IRCCS, 00149 Rome, Italy; carla.fontana@inmi.it

**Keywords:** *Arcanobacterium haemolyticum*, sinusitis, periorbital cellulitis, subdural empyema, cerebritis

## Abstract

*Arcanobacterium haemolyticum* is a facultative anaerobe, catalase-negative, Gram-positive pleomorphic rod, most commonly responsible for pharyngeal infections. Invasive *A. haemolyticum* infections are rare and typically involve immunocompromised patients; however, severe and invasive infections in immunocompetent patients have also been reported. Here we describe a case of sinusitis complicated by periorbital cellulitis, subdural empyema and cerebritis due to *A. haemolyticum* in an immunocompetent patient. The patient was initially treated with daptomycin + piperacillin/tazobactam and subsequently with linezolid + meropenem and required multiple surgical interventions to attain source control. Although uncommon, *A. haemolyticum* should be considered as a causative agent of severe infections complicating pharyngitis or sinusitis that may result from local extension or haematogenous spread, even in an immunocompetent host. We also present a literature review on central nervous system involvement by *A. haemolyticum* infection.

## 1. Introduction

*Arcanobacterium haemolyticum* is a facultative anaerobe, catalase-negative, Gram-positive pleomorphic rod, nonmotile and non-spore-forming, previously assigned to the genuses *Corynebacterium* and *Actinomycetes* [1]. It is most commonly responsible for pharyngeal infections, more frequently in the 15- to 25-year-old age group; clinical presentation is often similar to *Streptococcus pyogenes* infection, with fever, pharyngeal exudate, skin rash and cervical lymphadenopathy [2]. It is also implicated in skin and wound infections [3].

Invasive *A. haemolyticum* infections are rare and typically involve immunocompromised patients, causing peritonsillar abscess, bacteraemia, Lemierre syndrome, endocarditis, sinusitis and orbital cellulitis, brain abscess, pyogenic arthritis, osteomyelitis, empyema and pulmonary infiltrates with cavitation [4]. However, severe and invasive infections in immunocompetent patients have also been reported [5].

We present a rare case of *A. haemolyticum* central nervous system (CNS) infection in an immunocompetent individual, supplemented by a comprehensive review of the literature on this topic.

## 2. Case Report

**Figure 1:**
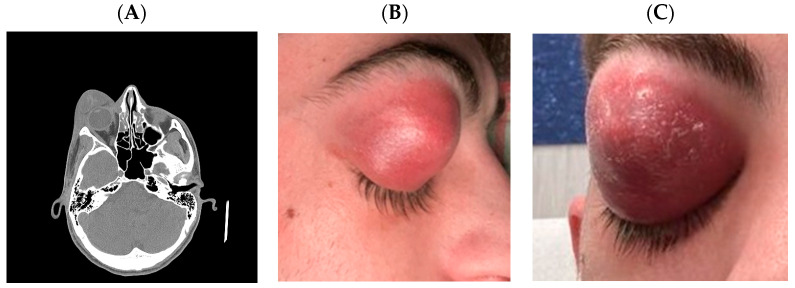
Right orbital cellulitis with periorbital abscess, proptosis, and associated sinusitis. (**A**) Head computed tomography scan that revealed a right orbital cellulitis with a large exophytic formation in the orbital and periorbital area inferiorly displacing the eyeball, together with right maxillary, frontal and ethmoidal sinusitis. (**B**,**C**) Patient’s pictures showing marked right periorbital edema, erythema and violaceous swelling causing complete mechanical ptosis and proptosis. The lesion appeared fluctuant, consistent with a subcutaneous abscess.

At the beginning of December 2023, a 21-year-old student nurse underwent a head CT scan due to a ten-day history of headache. He was diagnosed as having right fronto-maxillary sinusitis and started therapy with amoxicillin-clavulanate. Three days later, he developed right periorbital edema and the general practitioner suggested changing the antibiotic therapy to clarithromycin 500 mg every 12 h (bid), suspecting an allergic reaction. In a subsequent infectious diseases visit, the therapy was further changed to intramuscular ceftriaxone 2 g every 24 h (qd). On 18 December 2023, due to worsening of the local clinical picture, the patient went to the emergency room of a tertiary referral hospital in Rome (Italy) where he underwent blood cultures (which returned negative) and a head CT scan that revealed right orbital cellulitis with a large exophytic formation in the orbital and periorbital area inferiorly displacing the eyeball, together with right maxillary, frontal and ethmoidal sinusitis (Figure 1). The blood test showed the following: leucocyte 16,720 cells/mmc (polymorphonuclear 81%), Hb 16.2 mg/dL, platelets 372,000/mmc, C-reactive protein 10.4 mg/dL, aspartate aminotransferase (AST) 18 U/L, alanine aminotransferase (ALT) 15 U/L, glucose 101 mg/dL and creatinine 0.98 mg/dL. The HIV test was negative, glucose plasma levels were within normal limits, and no steroid or immunosuppressant exposure was reported around the time of presentation.

He started therapy with daptomycin 700 mg qd and piperacillin/tazobactam 4.5 gr every 6 h and was admitted to the maxillofacial surgery unit. A culture of drained material from periorbital and sinus collections grew *A. haemolyticum*, whose minimum inhibitory concentrations (MICs) were as follows: amoxicillin-clavulanate < 0.016 mg/L, azythromycin 2 mg/L, daptomycin 4 mg/L, linezolid 0.19 mg/L, meropenem 0.012 mg/L, piperacillin-tazobactam < 0.016 mg/L, clindamycin > 256 mg/L and metronidazole > 256 mg/L.

**Figure 2:**
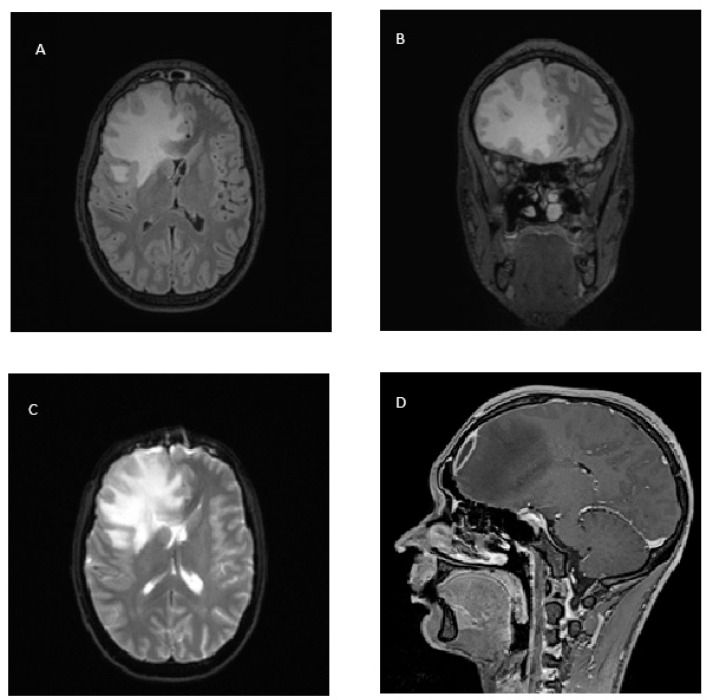
Magnetic resonance of the right frontal lobe showing cerebritis and abscess with ring enhancement and midline shift. (**A,B**) Axial and coronal fluid attenuated inversion recovery (FLAIR) images show an extensive hyperintense lesion in the right frontal lobe, with surrounding vasogenic edema and mass effect, consistent with cerebritis and abscess formation. (**C**) Axial diffusion-weighted imaging (DWI) images demonstrate marked hyperintensity within the lesion, indicative of restricted diffusion, characteristic of a pyogenic abscess. (**D**) Post-contrast T1-weighted image with fat saturation (sagittal view) reveals peripheral ring enhancement with central hypointensity, confirming the encapsulated nature of the abscess. Note the associated compression of the right lateral ventricle and midline shift.

On 8 January 2024, a cerebral magnetic resonance (MR) revealed the presence of a right frontal subdural purulent collection with a maximum thickness of 7 mm to be referred to as empyema; the frontal right cerebral tissue was also involved in the infection with a large edematous reaction shifting the midline structures 9 mm to the left (Figure 2). The patient was therefore transferred to the neurosurgery unit and on 9 January 2024, underwent right fronto-basal craniotomy, toilette of the right frontal sinus, drainage of the subdural frontal abscess collection and plastic reconstruction of the skull. The culture of intraoperative samples returned negative. On the same day, the antibiotic therapy was changed to intravenous linezolid 600 mg bid and meropenem 2 g every 8 h (tid); mannitol, dexamethasone and levetiracetam were also administered.

On 18 January 2024, the patient was transferred to our infectious diseases unit at “Spallanzani” hospital in Rome (Italy). On admission, he was in good general condition, alert, oriented and eupneic in room air. Antibiotic therapy was continued with meropenem 2 g tid until 3 February 2024, and linezolid 600 mg bid until 2 March 2024. Meropenem was withdrawn due to an increase in transaminase levels (AST 118 U/L, ALT 313 U/L), which subsequently decreased. Linezolid was well tolerated: on hospital discharge (2 March 2024), leucocytes were 3930/mmc, Hb 13.9 g/dL, platelets 211,000/mmc, AST 36 and ALT 83 U/L. The patient also received mannitol during the first 4 days, dexamethasone 8 mg bid with subsequent decalage and levetiracetam 500 mg bid (reduced to 500 mg qd on discharge).

**Figure 3:**
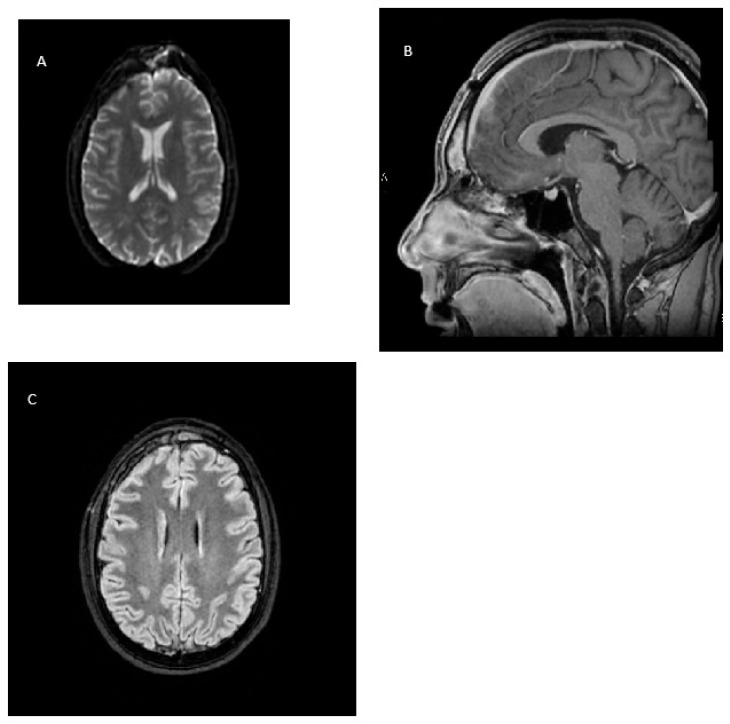
Magnetic resonance follow-up 40 days after surgical drainage and targeted antibiotic therapy. (**A**) DWI sequence reveals no evidence of restricted diffusion, confirming resolution of the abscess cavity. (**B**) 3D T1-weighted gradient echo demonstrates complete resolution of the previously observed ring-enhancing lesion in the right frontal lobe. (**C**) Axial FLAIR image shows normalization of signal intensity in the affected region, with no residual edema.

On 27 February 2024, a cerebral MR revealed a good reduction in the subdural collection at the craniotomy access and of the frontotemporal edema, with midline structures substantially in axis (Figure 3).

After a one-year follow-up, the patient is in stable general condition, without any recurrence of the disease, and is continuing his studies.

## 3. Discussion

*Arcanobacterium* species are Gram-positive, facultatively anaerobic bacilli that are often β-hemolytic and catalase-negative. The most clinically relevant species in humans is *A. haemolyticum.* It was first isolated in 1946 among patients with pharyngeal infections and skin rashes in the Pacific Islands [5,6]. It was formerly known as *Corynebacterium haemolyticum* but was reclassified as a separate genus in 1982 based on chemical and numerical phenetic data indicating that it was a distinct taxon worthy of generic status. A new genus, *Arcanobacterium*, was then described for the species *A. haemolyticum* [7]. Another species, *Arcanobacterium pyogenes* (now reclassified as *Trueperella pyogenes*), is primarily an animal pathogen but is implicated in rare zoonotic infections in humans, especially among individuals with close contact with livestock [8]. This organism is a normal inhabitant of the mucous membranes of domestic animals such as cattle, sheep and swine, and human infections have been reported in rural populations, often in those with underlying health conditions like cancer or diabetes [9].

*A. haemolyticum* is a Gram-positive (or gram-variable), non-motile, non-spore forming, catalase-negative, facultative anaerobic bacillus [5]. It grows well on blood- or serum-enriched medium, and its growth and haemolytic action are enhanced by the addition of carbon dioxide [5,6].

The identification of *A. haemolyticum* in clinical microbiology is challenging due to its inconspicuous colony morphology and its resemblance to other Gram-positive bacilli, particularly *Corynebacterium* species. On standard sheep blood agar, its β-hemolysis may be faint or delayed, often requiring the use of human or horse blood agar and incubation in a CO_2_-enriched atmosphere to enhance visibility. Morphologically, the organism appears as slender, curved rods that may be Gram-variable, further complicating differentiation from other coryneform bacteria. Traditional biochemical tests such as catalase and urease reactions offer limited specificity, and the reverse CAMP test, while helpful, is not definitive [9]. Consequently, advanced diagnostic tools such as matrix-assisted laser desorption/ionization time-of-flight mass spectrometry (MALDI-TOF MS) and 16S rRNA gene sequencing are becoming increasingly recommended for accurate identification. These methods provide species-level resolution and are particularly important in cases of invasive disease or when the organism is isolated from sterile body sites [10,11,12]. A more recent and relevant source of information on the microbiological challenges of identifying newly recognized pathogens is the MicrobeNet platform, available from the CDC (https://microbenet.cdc.gov, accessed on 5 June 2025). It provides access to MALDI-TOF MS spectra, 16S rRNA sequences and other reference data to assist with the identification of rare or emerging pathogens [13]. This could be particularly useful for organisms such as *A. haemolyticum*, which are often misidentified due to their subtle hemolytic patterns and morphological similarities to other coryneform bacteria. The use of advanced tools such as MALDI-TOF and sequencing is advised to overcome the limitations of traditional biochemical tests. The lack of rapid, specific diagnostic assays in many routine laboratories contributes to underdiagnosis and mismanagement, underscoring the need for heightened clinical awareness and tailored laboratory protocols. In the present case the identification of *A. haemolyticum* was achieved through a culture on blood agar under CO_2_-enriched conditions, followed by MALDI-TOF MS confirmation. The reverse CAMP test supported phenotypic identification. An anaerobic culture was performed to assess for coinfecting anaerobes, in line with best practices for polymicrobial CNS infections.

Humans are the principal reservoir, although animal infections have been described [14]. It can be found in the oropharynx of healthy people and is a common component of dental plaques; it can also be isolated from the respiratory tract of many animals, as well as from the soil [2]. No risk factors for infection have yet been identified; however, two different patient subsets are recognized: healthy young adults presenting with upper respiratory tract infections and older, often immunocompromised, patients presenting with skin and soft tissue infections [15]. *A. haemolyticum* may also be a rare cause of invasive disease, and some virulence factors could promote more aggressive infections. Phospholipase D, a ubiquitous enzyme, triggers a cascade, increasing local receptor concentration and enhancing bacterial adhesion to the pharyngeal epithelium. Arcanolysin, a cytolysin responsible for the germ’s haemolytic activity, facilitates cellular invasion. The ability of this bacterium to reside intracellularly has been hypothesized by Sayad and coll. as a cause of penicillin treatment failure [16]. Manifestations of *A. haemolyticum* invasive disease include severe sepsis, pulmonary abscess and pleural empyema, Lemierre’s syndrome, osteomyelitis, septic arthritis, spontaneous bacterial peritonitis, endocarditis and CNS infections. To date, we have retrieved from the medical literature 17 cases with CNS involvement (Table 1). We conducted a search in Pubmed using the keyword “*Arcanobacterium haemolyticum*” and we obtained 182 papers, among which 11 described cases with CNS infection. A further search with the keyword “*Corynebacterium haemolyticum*” retrieved 213 papers, among which 3 reported further and previously unidentified cases of CNS infection. The remaining three cases were found by examining the references of the papers reporting CNS involvement [1,2,5,15,17,18,19,20,21,22,23,24,25,26,27,28,29].

Although cases of isolated meningitis have been reported, the clinical history of most cases of CNS infection appear to evolve, as in this case, from sinusitis to subdural empyema, cerebritis and subsequent abscess formation necessitating surgical procedures. Anatomical pathways for bacterial entry into the CNS often involve hematogenous dissemination or direct extension from adjacent infected sites, such as the middle ear or paranasal sinuses. Data in the literature mention that adolescents carry a greater risk of rhinogenic intracranial complications because of the highly vascularized diploic venous system and development of the frontal sinuses in this age group [30]. In our patient, negative CNS cultures may reflect prior antibiotic exposure, low bacterial burden or viable but non-culturable organisms. Biofilm formation and sampling limitations may also contribute. The temporal and anatomical progression from sinusitis to CNS involvement, along with positive cultures from adjacent sites, supports *A. haemolyticum* as the etiologic agent.

Local extension of infection to CNS has also been described in relation to dental pathology, head trauma and upper respiratory tract infection [1,17,18,20,29]. In two cases, CNS involvement was the consequence of a mitral valve endocarditis with secondary septic emboli to the brain [15,22]: intracerebral hemorrhage complicated both cases, possibly caused by mycotic aneurysms.

As reported in Table 1, *A. haemolyticum* infection may occur alongside other microorganisms, most commonly *Fusobacterium necrophorum*, *Bacteroides* spp. and *Propionibacterium* spp. In particular, coinfection by A. *haemolyticum* and *F. necrophorum* has been reported in the majority of cases of polymicrobial infection, as detailed in a recent review of *A. haemolyticum* cases [31]. Factors underlying coinfection between these two bacteria and contributing to severe infections in some patients but not in others have not yet been defined [5]. A. *haemolyticum* and *F. necrophorum* infection has been described in cases of Lemierre’s syndrome (LS), a rare complication of oropharyngeal infection presenting with thrombophlebitis of the internal jugular vein with metastatic infective foci [32,33,34]. In our case, unfortunately, an ultrasound study of the neck veins was not performed, but the patient healed without anticoagulation, which renders LS unlikely.

CNS *A. haemolyticum* infection has been described in the context of EBV mononucleosis treated with prednisone, complicated by secondary bacterial sinusitis with intracranial extension leading to brain abscess and subdural empyema: EBV may have disrupted the upper respiratory tract epithelium, triggering secondary bacterial sinusitis with subsequent contiguous spread of infection to the brain in the setting of transient EBV-related immunosuppression exacerbated by prednisone therapy [19].

Clear indications for the optimal antibiotic treatment of *A. haemolyticum* infections are not available. This organism is usually resistant to trimethoprim-sulfamethoxazole, while most strains remain sensitive to clindamycin and macrolides [1,35]. Carlson et al. tested 138 clinical isolates of *A. haemolyticum* against different antibiotics: all strains were susceptible to phenoxymethylpenicillin, cephalosporins, erythromycin, azithromycin, clindamycin, vancomycin, doxycycline and ciprofloxacin, but were resistant to trimethoprim-sulfamethoxazole [36]. Penicillins have been the most frequently prescribed agents historically, and *A. haemolyticum* appears to be susceptible to many beta-lactams [1]. However, bactericidal tests suggested that some strains of *A. haemolyticum* may be tolerant to penicillins: Nyman et al. described high penicillin tolerance in 34 out of 40 *A. haemolyticum* isolates, suggesting that phenoxymethylpenicillin administration would be ineffective at eradicating *A. haemolyticum* from the pharynx [37]. On the other hand, microbiological eradication was obtained with erythromycin in 87% of patients, leading some authors to suggest macrolides as a primary treatment regimen [38]. Osterlund proposed that penicillin treatment failure could be due to the survival of intracellularly residing bacteria; in his study, erythromycin killed these bacteria [39]. Penicillins may therefore not be ideal as an initial monotherapy in serious infections caused by *A. haemolyticum*: previous authors on this topic have suggested the empiric combination of high-dose penicillin and erythromycin [1]. Our patient did not receive macrolides but was treated with daptomycin + piperacillin/tazobactam for 22 days, followed by linezolid + meropenem for 26 days; meropenem was then withdrawn due to an increase in liver enzymes and linezolid was continued for 28 more days. MICs of piperacillin/tazobactam, meropenem and linezolid against the strain of *A. haemolyticum* were <0.016 mg/L, 0.012 mg/L and 0.19 mg/L, respectively. Interpretation of these MICs are not available in official EUCAST and CLSI tables. Currently, no clinical breakpoints for *A. haemolyticum* are available in either CLSI or EUCAST guidelines [40,41]. This absence limits standardized interpretation of MIC values. EUCAST explicitly provides guidance for such cases, recommending that interpretation be based on pharmacokinetic/pharmacodynamic (PK/PD) data, achievable drug concentrations at the infection site, and published evidence. In the present case susceptibility testing was performed using broth microdilution according to CLSI guidelines. Quality control strains included *Staphylococcus aureus* ATCC 29213. In the absence of CLSI/EUCAST breakpoints for *A. haemolyticum*, MICs were interpreted based on PK/PD data and literature [35,40].

MICs of erythromycin and azithromycin against our strain were 2 mg/L for both drugs, much higher than the MIC_90_ of 0.06 mg/L described by Carlson and coll. for both drugs [36]. The MIC of daptomycin was 4 mg/L, and this value is difficult to interpret. A search in PubMed using the terms “*Arcanobacterium*” AND “daptomycin” retrieved zero results. Our patient received linezolid for 54 days, almost two times the suggested maximum duration of linezolid therapy. The companion beta-lactam drug (meropenem) had to be stopped due to side effects, and other beta-lactams were deemed not suitable in this situation to avoid similar undesirable consequences: for these reasons, linezolid was continued as a monotherapy. Fortunately, the drug was well tolerated, without drug-related toxicities. Veerman and coll. reported that 67 out of 78 patients with bone and joint infections were able to continue linezolid beyond 28 days, and 87% of them (58/67) completed therapy as scheduled, up to 12 weeks. Most of the adverse events attributable to linezolid occurred within 28 days of treatment initiation: if patients completed the first 28 days of therapy, the probability of a later occurrence of serious adverse events was low [42]. Some studies have suggested that therapeutic drug monitoring (TDM) of linezolid aimed at maintaining trough levels between 2 and 8 mg/L could help prevent thrombocytopenia and allow treatment to continue beyond 28 days [43,44]. In their recent experience, Kobayashi and coll. temporarily discontinued linezolid when platelet counts approached 60.000/mmc after the linezolid trough concentration reached 7.98 mg/L and obtained a regression of the toxicity based on TDM-guided linezolid dosing [44]. In our case TDM of linezolid was not performed, but the patient underwent blood cell counts two times per week and did not experience thrombocytopenia. Given the absence of breakpoints, the clinical relevance of MICs was assessed based on achievable serum and CNS drug levels. The linezolid MIC of 0.19 mg/L was significantly lower than its typical cerebrospinal fluid concentration (~3–7 mg/L), supporting its use in this CNS infection.

According to the antibiogram, potential alternatives to linezolid were meropenem (MIC 0.012 mg/L), amoxicillin/clavulanate (MIC < 0.016 mg/L), and piperacillin/tazobactam (MIC < 0.016 mg/L); however, meropenem had been withdrawn due to hepatic toxicity, and the last two drugs would have raised concerns regarding the passage of the blood–brain barrier.

To summarize, on imaging examinations, large paranasal/periorbital purulent collections or signs of CNS involvement should prompt surgical evaluation for timely evacuation and source control. Clear indications for *A. haemolyticum* antibiotic treatment are not available. The majority of the authors reporting *A. haemolyticum* CNS involvement used cephalosporins, aminopenicillins, penicillin G, vancomycin or metronidazole. Duration of treatment among cases that completed the therapy was most commonly 6 weeks or more.

## 4. Conclusions

Although uncommon, *A. haemolyticum* should be considered as a potential cause of severe infections that complicate pharyngitis or sinusitis which may result from local extension or haematogenous spread. This case shows the role of *A. haemolyticum* in a severe infection involving a young immunocompetent patient, which progressed from sinusitis to periorbital cellulitis, subdural empyema and cerebritis, and required multiple surgical interventions to attain source control. In light of the potentially life-threatening complications of this infection, a deeper knowledge surrounding *A. haemolyticum*, its identification in clinical microbiology, and its effective treatment is needed.

## Figures and Tables

**Table 1:** Cases of *A. haemolyticum* infection involving central nervous system.

Gender & Age	Organisms	Focus of Infection	Intracranial Complication	Antibiotic Treatment	Duration of Treatment	Ref.
F 16	*A.haemolyticum, Bacteroides *sp.*, Anaerococcus tetradius, Dialister micraerophilus, Erysipelotrichaceae *sp.*, Propionibacterium acnes*	Sinusitis, head trauma	Subdural empyema, cerebral abscess	Vancomycin, ceftriaxone, metronidazole, amoxicillin/clavulanate	6 weeks	[1]
M 17	*A.haemolyticum, Fusobacterium necrophorum*	Sinusitis	Meningitis, subdural empyema, cerebral edema	Vancomycin, cefepime, metronidazole	3 days (terminated due to fatal cerebral edema)	[2]
M 21	*A.haemolyticum, F. necrophorum, Streptococcus anginosus*	Pharyngitis and sinusitis	Subdural empyema, meningitis, cerebritis, cerebral abscess	Vancomycin, ceftriaxone, metronidazole, azithromycin, oral amoxicillin	6 weeks IV followed by 3 months oral	[5]
F 21	*A.haemolyticum*	Endocarditis	Cerebral abscess, intracerebral hemorrhage	Vancomycin, ceftriaxone, metronidazole, gentamicin	6 weeks	[15]
M 24	*A.haemolyticum, Propionibacterium avidum*	Pharyngitis, Sinusitis	Cerebral abscess	Ceftriaxone, metronidazole	7 weeks	[17]
M 18	*A.haemolyticum*	Dental extraction	Cerebral abscess	Ceftriaxone, penicillin G	4 weeks	[18]
M 20	*A.haemolyticum,* EBV	Sinusitis	Cerebral abscesses, subdural empyema, meningitis	Ceftriaxone, metronidazole	9 weeks	[19]
M 15	*A.haemolyticum,* *F. necrophorum*	Tonsillitis, sinusitis	Meningitis, abducent palsy	Cefotaxime, metronidazole	Not reported	[20]
M 58	*A.haemolyticum*	Not reported, immunocompromised	Meningitis	Teicoplanin, penicillin G, ceftazidime	16 days (discharge a.m.a.)	[21]
M 50	*A.haemolyticum*	Infective endocarditis	Cerebritis, cerebral abscess, intracerebral haemmorrhage	Penicillin G	7 days (fatal intracerebral hemorrhage)	[22]
M 24	*A.haemolyticum*	Sinusitis	Meningitis, abducent palsy	Gentamicin, ampicillin, metronidazole, probenecid	Not reported	[23]
M 11	*A.haemolyticum, bacteroides melanogenicus*	Not reported	Cerebral abscess	Penicillin G, metronidazole	Not reported	[24]
M 17	*A.haemolyticum, F. necrophorum*	Not reported	Meningitis, cerebral abscess, abducent palsy	Cephalothin, gentamicin, chloramphenicol	2 days (fatal cerebral abscess)	[25]
M 16	*A.haemolyticum*	Not reported	Cerebral abscess	Penicillin G	Not reported	[26]
M 65	*A.haemolyticum, B. fragilis*	Not reported	Meningitis, encephalitis	Penicillin, cloxacillin	Not reported	[27]
M 19	*A.haemolyticum*	Sinusitis, orbital cellulitis	Meningitis, cerebritis, intracranial collection, delayed cerebral ischemia	Not reported	40 days IV	[28]
M 54	*A.haemolyticum*	Poor dental conditions	Cerebral abscess	Amoxicillin, metronidazole	10 days IV + 3 weeks oral	[29]

EBV: Epstein–Barr virus; a.m.a.: against medical advice; IV: intravenously.

## Data Availability

The original contributions presented in this study are included in the article. Further inquiries can be directed to the corresponding author.
